# Healing through faith: spirituality and shrine visitation as coping mechanisms among haemophilia patients in Kashmir—an interpretative phenomenological study

**DOI:** 10.3389/frhs.2026.1802955

**Published:** 2026-09-15

**Authors:** Shabnam Mushtaq, Wakar Amin Zargar, Fayaz Ahmad Paul

**Affiliations:** 1 Department of Social Work, University of Kashmir, Srinagar, Jammu and Kashmir, India; 2 Department of Medical Social Service (Welfare) Unit, All India Institute of Medical Sciences (AIIMS), Rajkot, Gujarat, India; 3 Department of Social Work, Rajagiri College of Social Sciences (Autonomous), Kalamassery, Cochin, Kerala, India

**Keywords:** haemophilia, interpretative phenomenological analysis, Kashmir, qualitative research, religious coping, shrine visitation, spirituality

## Abstract

**Background:**

In Kashmir, Sufi traditions are integral to everyday life. Visits to shrines are a culturally significant expression of faith and may influence how individuals cope with lifelong health conditions such as haemophilia.

**Objective:**

This study sought to explore the experiences of adults living with haemophilia in Kashmir with spirituality and shrine visitation as coping methods to deal with the psychological, emotional and social challenges of their condition.

**Methods:**

A qualitative study using the Interpretative Phenomenological Analysis (IPA) approach was conducted at the Haemophilia day care centre, Sri Maharaja Hari Singh (SMHS) Hospital, Srinagar, Jammu and Kashmir, India. Twelve adults (aged ≥18 years) diagnosed with Haemophilia A or Haemophilia B were recruited through purposive sampling. Data were collected using semi-structured, in-depth interviews and analysed following the principles of IPA.

**Results:**

Analysis revealed one overarching theme, *visiting shrines as a spiritual coping mechanism,* comprising three interconnected subthemes: *(1) changing patterns of shrine visitation across the life course, (2) belief in God and living with haemophilia, and (3) haemophilia as God's will.* Participants reported visiting shrines as a source of hope, emotional comfort, inner peace, resilience and spiritual connection. Participants recognized the importance of biomedical treatment but also saw shrine visitation as a complementary coping strategy of meaning, strengthening faith and facilitating acceptance of their illness. Participants also found that religious beliefs allowed them to reinterpret suffering through trust in God, which contributed to their psychological resilience when viewed as a lifelong condition.

**Conclusion:**

Spirituality and shrine visits are important culturally embedded coping mechanisms for people living with haemophilia in Kashmir. These practices are not a substitute for medical treatment but are a complement to conventional healthcare in addressing the emotional, existential and psychosocial needs of patients. Integrating culturally sensitive spiritual care into haemophilia services may contribute to more holistic, person-centred care.

## Introduction

Haemophilia is a lifelong inherited bleeding disorder caused by a deficiency of clotting factor VIII (Haemophilia A) or factor IX (Haemophilia B) (1). It is a genetic X-linked disease affecting mainly males and characterized by recurrent spontaneous or prolonged bleeding episodes mainly in joints and muscles leading to chronic pain, progressive arthropathy, physical disability and decreased quality of life (2–5). Haemophilia is associated not only with physical complications, but also with significant psychosocial and economic burdens for the individual and their families. Besides medical treatment, constant adjustment and long-term coping are continuously required for these patients. Coping is defined as the cognitive and behavioral effort to manage the demands of stressful situations that are appraised as taxing or exceeding one's resources (6). Better psychological well-being, treatment adherence, resilience and health-related quality of life have been associated with successful coping methods in people with chronic illness (7). In addition to medical treatment, people rely on personal, social and cultural resources to manage the uncertainty and distress of chronic disease.

Spirituality and religion are some of the most widely recognised coping resources in chronic illness. Spirituality is commonly understood as a person's search for meaning, purpose and connection with the sacred, whereas religion is an organized system of beliefs, rituals and practices through which spirituality is often expressed (8, 9). Although conceptually distinct, the two often overlap in everyday life and both contribute to psychological adjustment, hope, resilience and mental health (10, 11). Prayer, pilgrimage, worship and charity have been shown to provide emotional comfort, acceptance and reduced psychological distress among people living with chronic illnesses (12–14). Within the cultural context of Kashmir spirituality is interwoven with the Islamic and Sufi traditions. Kashmir valley has a large number of Sufi shrines (*Ziyarat*) which are important sites for religious worship, spiritual reflection and community support (15). These shrines are visited for Barakah (*blessings*), Mannat (*vow fulfillment*), prayers, thanksgiving and solace in sickness and hardship which is considered as culturally important activity (16). Thus, religion and spirituality are rooted in the local socio-cultural context and shrine visitation is one of the major manifestations of both faith and coping (17).

Spirituality may provide emotional support to people with haemophilia and may help them to understand living with a lifelong condition associated with recurrent pain, physical limitation and uncertainty. Spiritual and religious coping has been associated with better psychological well-being among those living with chronic diseases such as cancer, HIV/AIDS, diabetes and chronic pulmonary diseases (8, 12, 13, 18). Religious practices such as prayer, meditation and spiritual connectedness have been associated with increased resilience and reduced levels of psychological distress (10, 19). Although spirituality is a recognized dimension of health care, the impact of spirituality on the lives of people with haemophilia is unknown (20). Most of the studies on haemophilia have been on disease pathology, treatment outcomes, quality of life and psychological functioning but relatively less on spirituality and religious coping (21). Moreover, most of the previous research has adopted a quantitative approach to measure spirituality using standardized measurement tools and has not provided significant insights into how people perceive and experience spirituality in their cultural and religious contexts (22–24).

This ignorance is most marked in Kashmir where pilgrimage to shrines is part of everyday religious and cultural life. Kashmiri shrines have been studied in the past from historical, cultural and anthropological perspectives but their role in coping with chronic illness has received limited research attention (15, 17). Similarly, studies on the lived experiences of people living with haemophilia in Kashmir are also limited. Thus, the present study adopted Interpretative Phenomenological Analysis (IPA) to explore the lived experiences of adult persons with haemophilia in Kashmir on spirituality and shrine visitation as coping mechanisms. By examining participants' personal narratives, this study aims to offer a culturally informed understanding of the role of spirituality, faith and shrine visitation in resilience, meaning-making and psychosocial adaptation in the setting of living with haemophilia.

## Materials and methods

### Research design

The present study adopted a qualitative research design, utilizing Interpretative Phenomenological Analysis (IPA) to explore the lived experiences of people with haemophilia concerning spirituality and visiting shrines as a coping strategy. IPA is underpinned by phenomenology, hermeneutics and idiography and is particularly appropriate for exploring how people interpret and make sense of their personal experiences (25).

### Study setting

The study was conducted at the Haemophilia Day Care Centre of Sri Maharaja Hari Singh (SMHS) Hospital, a tertiary care public healthcare institution located in Srinagar, Jammu and Kashmir, India. The centre provides specialised clinical care and follow-up services for individuals living with haemophilia across the Kashmir Valley.

### Participants and sampling

Participants were recruited using purposive sampling to obtain rich and information-rich accounts relevant to the study objectives. Eligible participants were adults (≥18 years) diagnosed with Haemophilia A (Factor VIII deficiency) or Haemophilia B (Factor IX deficiency) and registered at the Haemophilia Day Care Centre. Individuals younger than 18 years, those diagnosed with other inherited bleeding disorders, and those unwilling to provide informed consent were excluded. The study involved 12 participants in total. Recruitment continued until data saturation was reached, with no new concepts identified in subsequent interviews. The sample size is in line with recommendations for IPA studies, which tend to have small and relatively homogenous samples to allow detailed idiographic analysis (26–28).

### Data collection

Data were collected through semi-structured in-depth interviews by the first author at Haemophilia Day Care Centre during 16 July 2022 to 27 September 2022. An interview guide was developed based on the objectives of the study and the relevant literature. Questions investigated participants' lived experiences with haemophilia, perceptions of spirituality, views on shrine visitation and the role of religious practices in coping with their illness. Broad, open-ended questions started the interview, such as “*How has haemophilia impacted on your life?, “How does spirituality factor into your life?”, “How do you perceive shrine visitation?”, “Has your faith influenced the way you cope with your illness?”*

Interviews were conducted for approximately 40–50 min and were audio-recorded with the written informed consent of participants. Field notes were taken throughout the data collection process to document non-verbal expressions, contextual observations and researchers’ reflections in an effort to enhance contextual understanding. Although the interviews were completed in 2022, the subsequent stages of verbatim transcription, coding, iterative analysis, interpretation, manuscript preparation, and revision required considerable time before submission. Such timelines are common in qualitative research because of the depth and iterative nature of data analysis.

### Data analysis

Audio-recorded interviews were transcribed verbatim and analysed using the IPA framework proposed by Pietkiewicz and Smith (2014) (27). Analysis involved repeated reading of transcripts, detailed initial noting, development of emergent themes and clustering of related themes into higher-order conceptual categories. Each transcript was analysed independently to preserve the idiographic focus of IPA before patterns of convergence and divergence were examined across participants. The analytical process was iterative, recognising the double hermeneutic characteristic of IPA, whereby participants interpreted their lived experiences while the researchers interpreted participants' accounts. To enhance the trustworthiness of the analysis, reflexive notes were maintained throughout the analytical process to acknowledge researchers' assumptions and minimise interpretative bias. Representative verbatim quotations were incorporated to illustrate each theme and to ensure that the findings remained grounded in participants' lived experiences.

## Ethical considerations

Ethical approval was obtained from the Institutional Review Board of Government Medical College, Srinagar, affiliated with SMHS Hospital. Written informed consent was obtained from all participants before data collection. Participants were informed about the objectives of the study, the voluntary nature of participation, their right to withdraw at any stage without consequence, and the confidentiality of their information. All interview recordings and transcripts were anonymised and stored securely to protect participants' privacy.

## Results

Twelve adults diagnosed with haemophilia participated in the study. Participants ranged in age from 18 to 60 years. The majority were male (*n* = 10, 83.3%), while two participants (16.7%) were female. Nine participants (75.0%) were married and three (25.0%) were unmarried. Most participants had Haemophilia A (*n* = 11, 91.7%), whereas one participant (8.3%) had Haemophilia B. Regarding disease severity, six participants (50.0%) had severe haemophilia, five (41.7%) had moderate haemophilia, and one (8.3%) had mild haemophilia. Educational attainment varied, with seven participants (58.3%) having no formal education, three (25.0%) completing primary education, and two (16.7%) completing higher secondary education (Table 1).

**Table 1:** Sociodemographic characteristics of the study participants (*n* = 12*).*

Variables	*N*	Percentage%
Gender		
Male	10	83.3%
Female	02	16.7%
Educational status		
Illiterate	07	58.3%
Primary level	03	25.0%
Higher secondary level	02	16.7%
Marital status		
Married	09	75.0%
Unmarried	03	25.0%
Type of haemophilia		
Haemophilia A	11	91.7%
Haemophilia B	01	8.3%
Severity of the disease		
Severe	06	50.0%
Moderate	05	41.7%
Mild	01	8.3%

### Overview of themes

Interpretative Phenomenological Analysis identified one overarching theme, “*Shrine Visitation as a Spiritual Coping Mechanism,”* comprising three interrelated subthemes:

1. Changing Patterns of Shrine Visitation Across the Life Course
2. Belief in God and Living with Haemophilia
3. Haemophilia as God's Will

These themes illustrate how spirituality and religious beliefs shaped participants' understanding of haemophilia and influenced their coping with the emotional, psychological, and social challenges associated with the condition.

### Overarching theme: shrine visitation as a spiritual coping mechanism

Participants consistently described shrine visitation as an important source of emotional comfort, hope, and spiritual strength. Although all participants recognised the importance of medical treatment, many regarded visits to Sufi shrines as a complementary practice that helped them cope with the emotional burden of living with haemophilia. For several participants, shrine visitation provided a sense of peace during periods of physical suffering and emotional distress. “*My life feels like that of a prisoner. Because of my physical limitations, I cannot always offer prayers in the mosque. Whenever I visit a Sufi shrine, I experience inner peace and emotional comfort”* (Participant 2). Participants frequently described shrines as places where they experienced hope and psychological reassurance rather than expecting miraculous healing. “*Whenever I visit the shrine, I feel peaceful and return with renewed hope that one day I may recover”* (Participants 4 and 7). Another participant explained: “*Whenever I feel lonely or emotionally overwhelmed, I visit Makhdoom Sahib. I believe there is a divine presence there that gives me strength to continue my struggle”* (Participant 5).

Several participants also visited shrines before hospital appointments or during periods of increased stress. “*Before going to the hospital, I often visit a shrine. I know it will not cure my illness, but it gives me strength to face whatever lies ahead”* (Participant 4). For some, shrine visitation was closely associated with concerns about family and future generations. “*When my wife was expecting our first child, I visited the shrine every day, praying that my child would not inherit this disease”* (Participant 6). Participants also described financial hardship associated with lifelong treatment, during which spirituality provided reassurance and hope. “*Life is full of uncertainty and financial difficulties, but with God's grace and the blessings of the shrine, I have been able to continue managing my treatment”* (Participant 12). Overall, shrine visitation emerged as a culturally meaningful coping practice that fostered emotional support, hope, inner peace, and spiritual connectedness while living with a chronic illness (Table 2).

**Table 2:** Representative quotations illustrating the overarching theme and three interrelated subthemes identified through interpretative phenomenological analysis.

Overarching theme/Subtheme	Participant(s)	Representative participant comment
Overarching Theme: Shrine Visitation as a Spiritual Coping Mechanism	Participant 2	*“My life feels like that of a prisoner. Because of my physical limitations, I cannot always offer prayers in the mosque. Whenever I visit a Sufi shrine, I experience inner peace and emotional comfort.”*
Overarching Theme: Shrine Visitation as a Spiritual Coping Mechanism	Participants 4 and 7	*“Whenever I visit the shrine, I feel peaceful and return with renewed hope that one day I may recover.”*
Overarching Theme: Shrine Visitation as a Spiritual Coping Mechanism	Participant 5	*“Whenever I feel lonely or emotionally overwhelmed, I visit Makhdoom Sahib. I believe there is a divine presence there that gives me strength to continue my struggle.”*
Overarching Theme: Shrine Visitation as a Spiritual Coping Mechanism	Participant 4	*“Before going to the hospital, I often visit a shrine. I know it will not cure my illness, but it gives me strength to face whatever lies ahead.”*
Overarching Theme: Shrine Visitation as a Spiritual Coping Mechanism	Participant 6	*“When my wife was expecting our first child, I visited the shrine every day, praying that my child would not inherit this disease.”*
Overarching Theme: Shrine Visitation as a Spiritual Coping Mechanism	Participant 12	*“Life is full of uncertainty and financial difficulties, but with God's grace and the blessings of the shrine, I have been able to continue managing my treatment.”*
Subtheme 1: Changing Patterns of Shrine Visitation Across the Life Course	Participant 3	*“During my younger years, I visited the shrine every week, believing that God might cure my illness. Even today, I occasionally visit these places for inner peace.”*
Subtheme 1: Changing Patterns of Shrine Visitation Across the Life Course	Participant 8	*“Earlier I visited many shrines, but as I grew older I realised that this disease cannot be cured. I have to live with it, and only God knows what is best.”*
Subtheme 1: Changing Patterns of Shrine Visitation Across the Life Course	Participants 7 and 12	*“When I was a child, my parents took me to different shrines whenever I became ill. With time I realised that healing ultimately comes from God. I still respect these shrines, but I no longer feel the need to visit them regularly.”*
Subtheme 1: Changing Patterns of Shrine Visitation Across the Life Course	Participant 11	*“My mother used to take me to shrines every Thursday believing that I would recover, but nothing changed.”*
Subtheme 2: Belief in God and Living with Haemophilia	Participant 2	*“I never miss my prayers because God is my greatest support.”*
Subtheme 2: Belief in God and Living with Haemophilia	Participant 7	*“Whatever I do—whether travelling, studying, or working—I always remember God.”*
Subtheme 2: Belief in God and Living with Haemophilia	Participants 8 and 12	*“I have placed my trust in God. Hopefully, He will heal us one day.”*
Subtheme 2: Belief in God and Living with Haemophilia	Participant 8	*“I always pray that my children will be free from this disease. Whatever happens, I trust God completely.”*
Subtheme 3: Haemophilia as God's Will	Participant 5	*“God loves us, which is why He has chosen us to face this challenge.”*
Subtheme 3: Haemophilia as God's Will	Participants 10 and 12	*“I have experienced poverty, suffering, and illness throughout my life, but I never complain. I believe God knows what is best, and this is a test of my faith.”*
Subtheme 3: Haemophilia as God's Will	Participants 7 and 9	*“I never questioned God's decision. Since developing this illness, I have become much closer to Him.”*
Subtheme 3: Haemophilia as God's Will	Participant 5	*“When I was first diagnosed, I was frightened and confused. Gradually, I came to believe that God had given me this illness to make me stronger, and that belief brought me peace.”*
Subtheme 3: Haemophilia as God's Will	Participant 1	*“Sometimes I think this illness is a punishment for my mistakes. I ask God to forgive me if I have done anything wrong.”*

#### Subtheme 1: changing patterns of shrine visitation across the life course

Participants described changes in their shrine-visiting practices over time. Younger participants generally reported visiting shrines more frequently, often hoping for recovery or divine intervention. One participant reflected: “*During my younger years, I visited the shrine every week, believing that God might cure my illness. Even today, I occasionally visit these places for inner peace”* (Participant 3). In contrast, several older participants reported that their understanding of haemophilia evolved with age. Although they continued to respect shrines, they no longer believed that shrine visitation alone could cure the disease. As one participant explained: “*Earlier I visited many shrines, but as I grew older I realised that this disease cannot be cured. I have to live with it, and only God knows what is best”* (Participant 8).

Another participant stated: “*When I was a child, my parents took me to different shrines whenever I became ill. With time I realised that healing ultimately comes from God. I still respect these shrines, but I no longer feel the need to visit them regularly”* (Participants 7 and 12). Similarly, another participant recalled: “*My mother used to take me to shrines every Thursday believing that I would recover, but nothing changed”* (Participant 11). These narratives indicate that while shrine visitation remained culturally meaningful throughout life, its purpose gradually shifted from seeking physical healing to finding emotional comfort, acceptance, and spiritual reassurance.

#### Subtheme 2: belief in God and living with haemophilia

Participants consistently described faith in God as central to their experience of living with haemophilia. Their narratives reflected three closely connected dimensions: hope, trust, and acceptance. One participant explained: “*I never miss my prayers because God is my greatest support”* (Participant 2). Another participant described maintaining a constant spiritual connection with God. “*Whatever I do—whether travelling, studying, or working—I always remember God”* (Participant 7). Several participants expressed complete trust that God would guide and support them despite the uncertainty of their illness. “*I have placed my trust in God. Hopefully, He will heal us one day”* (Participants 8 and 12). For many participants, faith also influenced decisions about marriage and parenthood. “*I always pray that my children will be free from this disease. Whatever happens, I trust God completely”* (Participant 8). Faith therefore emerged as an important psychological resource that provided hope, reduced fear, and enabled participants to cope with the lifelong challenges associated with haemophilia.

#### Subtheme 3: haemophilia as God's will

Participants commonly interpreted haemophilia as part of God's will or as a test of faith. Viewing the illness through this perspective enabled many participants to accept their condition and find meaning despite ongoing physical suffering. One participant stated: “*God loves us, which is why He has chosen us to face this challenge”* (Participant 5). Another participant reflected: “*I have experienced poverty, suffering, and illness throughout my life, but I never complain. I believe God knows what is best, and this is a test of my faith”* (Participants 10 and 12). Some participants explained that their illness strengthened their relationship with God. “*I never questioned God's decision. Since developing this illness, I have become much closer to Him”* (Participants 7 and 9).

One participant described how acceptance developed over time. “*When I was first diagnosed, I was frightened and confused. Gradually, I came to believe that God had given me this illness to make me stronger, and that belief brought me peace”* (Participant 5). A few participants interpreted haemophilia as a consequence of their past actions and viewed it as an opportunity for repentance. “*Sometimes I think this illness is a punishment for my mistakes. I ask God to forgive me if I have done anything wrong”* (Participant 1). Although participants interpreted the meaning of haemophilia differently, their narratives consistently demonstrated that faith helped them understand their illness, accept uncertainty and maintain hope while living with a lifelong condition.

## Discussion

This study explored the lived experiences of adults with haemophilia in Kashmir regarding spirituality and shrine visitation as coping mechanisms. The findings suggest that spirituality plays a significant role in helping individuals cope with the emotional, psychological and existential challenges associated with a lifelong chronic illness. Participants described shrine visitation, faith in God and acceptance of illness as important sources of hope, resilience and meaning. A key finding was that shrine visitation served as a complementary coping strategy rather than an alternative to medical treatment. Participants recognised the importance of biomedical care while describing Sufi shrines as places that offered emotional comfort, inner peace and psychological strength during periods of illness. These findings are consistent with Pargament's theory of religious coping, which suggests that religious beliefs and practices help individuals interpret stressful experiences and maintain hope during adversity (13, 29). Similar observations have been reported among individuals living with chronic illnesses, where spirituality has been associated with improved psychological well-being, resilience and quality of life (8, 12, 18).

Another important finding was the evolution of shrine-visiting practices across the life course. Younger participants often visited shrines hoping for recovery or divine intervention, whereas older participants increasingly accepted haemophilia as a lifelong condition requiring medical management. Although regular shrine visitation declined with age, participants continued to engage in religious practices such as prayer and remembrance of God. This finding suggests that spiritual coping is dynamic and evolves as individuals gain greater understanding and acceptance of their illness. Faith in God emerged as another important coping resource. Participants described trust in God, regular prayer, and spiritual connectedness as helping them manage fear, uncertainty and emotional distress. These findings support previous research demonstrating that positive religious coping promotes resilience and psychological adjustment among people living with chronic illnesses (8, 19). For many participants, faith provided reassurance and encouraged optimism despite the lifelong nature of haemophilia.

Participants also commonly interpreted haemophilia as God's will or as a test of faith. Viewing the illness through this perspective enabled many to develop acceptance, patience, and meaning despite ongoing physical suffering. Similar findings have been reported in studies showing that spiritual beliefs facilitate meaning-making and emotional adjustment in chronic illness (8, 13). However, a few participants perceived their illness as punishment for past actions, reflecting negative religious coping. Although these beliefs were associated with guilt, they were often accompanied by hope for forgiveness and renewed spiritual commitment. The findings should be understood within the socio-cultural context of Kashmir, where Sufi shrines occupy an important place in religious and community life (15, 17). Participants viewed shrine visitation not merely as a religious ritual but as a culturally meaningful practice that strengthened hope, emotional well-being, and social connectedness. This highlights the importance of understanding spirituality within its cultural context rather than viewing it as a universal construct.

The present study has important clinical implications. Healthcare professionals working with individuals living with haemophilia should recognise that spirituality may serve as an important psychological resource that complements medical treatment. Incorporating culturally sensitive discussions about patients' spiritual beliefs into psychosocial care may strengthen therapeutic relationships, promote resilience, and support holistic, person-centred care. Overall, this study contributes to the limited qualitative literature on spirituality among people living with haemophilia. It demonstrates that shrine visitation, faith in God, and spiritual meaning-making are important coping resources that help individuals adapt to the psychological and social challenges of living with a lifelong chronic illness.

### Recommendations

The findings highlight the importance of incorporating culturally sensitive and spiritually informed care into the management of haemophilia. Healthcare professionals should acknowledge patients' spiritual beliefs and practices while reinforcing adherence to evidence-based medical treatment. Psychosocial interventions may incorporate discussions on spirituality, where appropriate, to enhance emotional resilience and coping. Collaboration between healthcare providers, mental health professionals, and community faith leaders may further support holistic and person-centred care.

### Strengths of the study

This study provides novel qualitative evidence on the role of spirituality and shrine visitation as coping mechanisms among individuals living with haemophilia in Kashmir. Using Interpretative Phenomenological Analysis enabled an in-depth exploration of participants' lived experiences within their socio-cultural context. The study contributes to the limited literature on spirituality in haemophilia by demonstrating how religious beliefs, faith, and shrine visitation facilitate resilience, meaning-making, and psychosocial adaptation to a lifelong chronic illness.

### Limitations of the study

The study has several limitations. First, the small sample drawn from a single tertiary care centre may limit the transferability of the findings to other settings. Second, purposive sampling may have resulted in the inclusion of participants who were more spiritually inclined. Third, the findings are based on self-reported experiences and may be influenced by recall and social desirability biases. Finally, the study did not examine the relationship between spirituality and objective clinical outcomes, limiting conclusions regarding its impact on disease management.

### Future directions

Future research should employ longitudinal and mixed-method designs to examine how spirituality influences psychological well-being, treatment adherence, and quality of life among individuals living with haemophilia. Comparative studies across different cultural and religious settings would improve understanding of the influence of context on spiritual coping. Further research should also evaluate the effectiveness of integrating culturally sensitive spiritual care into multidisciplinary haemophilia services.

## Conclusion

This study demonstrates that spirituality and shrine visitation are important coping resources for adults living with haemophilia in Kashmir. Participants viewed shrine visitation, faith in God, and acceptance of illness as sources of hope, emotional strength, and meaning while living with a lifelong condition. Rather than replacing biomedical treatment, these practices complemented conventional healthcare by addressing emotional and existential needs. The findings underscore the importance of culturally sensitive, person-centred care that recognises patients' spiritual beliefs alongside medical and psychosocial interventions.

## Data Availability

The original contributions presented in the study are included in the article/Supplementary Material, further inquiries can be directed to the corresponding author.
